# 3,6-Dihydroxyflavone regulates microRNA-34a through DNA methylation

**DOI:** 10.1186/s12885-017-3638-1

**Published:** 2017-09-05

**Authors:** Xiaoli Peng, Hui Chang, Junli Chen, Qianyong Zhang, Xiaoping Yu, Mantian Mi

**Affiliations:** 1 Research Center for Nutrition Correspondence and Food Safety, Third Military Medical University, Chongqing Key Laboratory of Nutrition and Food Safety, 30 Gaotanyan Street, Shapingba District, Chongqing, 400038 China; 2 Department of Public Health, School of Preclinical Medicine, Chengdu Medical College, Chengdu, China

**Keywords:** Breast cancer, Carcinogenesis, 3,6-Dihydroxyflavone, TET1, DNMT1, miR-34a, Methylation

## Abstract

**Background:**

Breast cancer is the common cancer in China. In previous study, we determined that 3,6-dihydroxyflavone (3,6-DHF) increases miR-34a significantly in breast carcinogenesis, but the mechanism remains unclear.

**Methods:**

We used qRT-PCR to analyze miR-34a and ten-eleven translocation (TET)1, TET2, TET3 levels in breast cancer cells. With a cellular breast carcinogenesis model and an experimental model of carcinogenesis in rats, TET1 levels were evaluated by western blot analysis and immunofluorescence. TET1 and 5hmC (5-hydroxymethylcytosine) levels were evaluated by immunofluorescence in nude mouse xenografts of MDA-MB-231 cells. Chromatin immunoprecipitation(ChIP) assayed for TET1 on the TET1 promoter, and dot blot analysis of DNA 5hmC was performed in MDA-MB-231 cells. We evaluated the mechanism of 3,6-DHF on the expression of tumor suppressor miR-34a by transfecting them with DNA methyltransferase (DNMT)1 plasmid and TET1 siRNA in breast cancer cells. Methylation-specific PCR detected methylation of the miR-34a promoter.

**Results:**

First, we found that 3,6-DHF promotes the expression of TET1 during carcinogen-induced breast carcinogenesis in MCF10A cells and in rats. 3,6-DHF also increased TET1 and 5hmC levels in MDA-MB-231 cells. Further study indicated that TET1 siRNA and pcDNA3/Myc-DNMT1 inhibited the 3,6-DHF reactivation effect on expression of miR-34a in breast cancer cells. Methylation-specific PCR assays indicated that TET1 siRNA and pcDNA3/Myc-DNMT1 inhibit the effect of 3,6-DHF on the demethylation of the miR-34a promoter.

**Conclusions:**

Our study showed that 3,6-DHF effectively increases TET1 expression by inhibiting DNMT1 and DNA hypermethylation, and consequently up-regulates miR-34a in breast carcinogenesis.

## Background

Breast cancer is a common cancer and the leading cause of cancer deaths in China [1]. Current chemotherapy treatments for breast cancer cause serious side effects; plant-based bioactive compounds are desired as chemotherapeutic drugs in cancer treatment due to their minimal side effects. Dietary flavonoids have been identified for cancer therapy and prevention because of their ability to suppress cancer cell proliferation [2], induce cell-cycle arrest and promote apoptosis [3]. In our previous experiment, we have identified that 3,6-DHF has the effect to inhibit breast carcinogenesis [4]. In the present study, we investigate the mechanism of 3,6-DHF’s anti-carcinogenesis property in the context of breast carcinogenesis.

Phytochemicals extracted from plants may modulate and reverse gene transcription, aberrant epigenetic changes, including DNA methylation, histone modification and non-coding RNA (miRNA) alteration [5]. DNA methylation change patterns can occur throughout the life of an individual; some changes can be a physiological response to environmental changes, whereas others might be associated with a pathological process such as oncogenic transformation [6]. DNA methylation dysregulation contribute to silencing tumor suppressor genes or activating oncogenes in tumor progression [7, 8]. DNA methyltransferases (DNMTs) play key roles in epigenetic methylation of DNA. DNMTs overexpression results in hypermethylation and DNMT1 deletion leads to DNA demethylation [9]. The ten-eleven translocation (TET) family (TET1/2/3) are Fe(II)- and 2-oxoglutarate (2OG)-dependent dioxygenases that convert 5-methylcytosine to 5-hydroxymethylcytosine(5hmC), and play potential roles in epigenetic through DNA demethylation [10]. Dysfunction of TET and DNMT activity is considered an epigenetic hallmark of human cancers [11, 12]; Disruption in DNA methylation and demethylation dynamics is intimately implicated in carcinogenesis [13]. Our previous research found that 3,6-DHF inhibits DNMT1 effectively. We propose that 3,6-DHF would have an effect on the balance of methylation and demethylation in breast carcinogenesis and breast cancer cells.

DNA hypermethylation is a major epigenetic event which is associated with tumor suppressor gene silencing. MiR-34a is a miRNA regulated by the p53 network at the transcriptional level and has been shown to be remarkably down regulated in a variety of cancers. Research shows that the miR-34a promoter hypermethylation leads to its epigenetic inactivation [14–17]. MiR-34a may counteract the p53 response to DNA damage [18] and miR-34a hypermethylation occurs in pre-cancerous lesions in tumor formation [19]. Upregulating miR-34a changes its target genes expression involving in multiple signal transduction pathways, represses tumor growth significantly [20, 21], and may be an efficient strategy for cancer treatment. In our previous research, we observed that 3,6-DHF up-regulates the miR-34a and over-expressed miR-34a promoted cytotoxicity and apoptosis in breast cancer cells induced by 3,6-DHF [22]. In this paper, we explored how DNA methylation and demethylation influence the effect of 3,6-DHF on miR-34a.

In this paper, we demonstrate that 3,6-DHF demethylases the miR-34a promoter by inhibiting DNMT1 activity and increasing TET1 expression. We also show that 3,6-DHF increases TET1 expression partially by inhibiting the activity of DNMT1. These results suggest that 3,6-DHF can modulate the expression of anticancer genes by regulating the imbalance of DNA methylation and demethylation. Furthermore, our findings provide a novel epigenetic mechanism contributing to breast cancer chemoprevention by flavonoids.

## Methods

### Chemicals and reagents

3,6-DHF was purchased from Alfa Aesar (Massachusetts, US); FBS and DMEM/F12 medium were from HyClone (Beijing, China); Trizol reagent, Lipofectamine 2000, gentamicin, insulin, Opti-Mem and horse serum were from Invitrogen (Carlsbad, CA, USA); all antibodies were from Cell Signaling Technology (Danvers, MA, USA). 4-(methylnitrosamino)-1-(3-pyridyl)-1-butanone (NNK), benzo[a]pyrene (B[a]P), 1-methyl-1-nitrosourea (MNU) and other chemicals were from Sigma-Aldrich (St. Louis, MO, USA). The pcDNA3/Myc-DNMT1 (Plasmid 36,939) plasmid was provided by Addgene (MA, USA). TET1 siRNA(sc-154,204) was from Santa Cruz Biotechnology. The cell lines were obtained from the Institute of Biochemistry and Cell Biology, Chinese Academy of Sciences (Shanghai, China).

### Animals and treatment

Mammary gland and tumor samples used in this study were obtained in previously published carcinogenesis and cancer cell grafting experiments. Animal experiments performed as previously described [22]. BALB/c nude mice (aged 42–48 days, 15–20 g) and Female Sprague–Dawley (SD) rats (aged 42–48 days, 145–165 g) were bred and maintained in accordance with our institutional guidelines. All of the animal procedures were approved by the Animal Ethics Committee of the Third Military Medical University. **Experimental model of carcinogenesis in rats:** Rat carcinogenesis model was established as previously described [22]. The rats were fed 3,6-DHF (20 mg/kg/day; *n* = 12) in the 3,6-DHF administration group,fed the vehicle alone in the control group. All rats were injected MNU (50 mg/kg). The rats were sacrificed at the end of the experiment. **Xenograft in nude mice:** Female BALB/c nude mice were implanted with MDA-MB-231 cells at a density of 2 × 10^6^ cells/ml s.c. into the right axilla, and randomly divided into the control(normal saline; *n* = 6) and 3,6-DHF administration groups(20 mg/kg/day; *n* = 6). Mice were sacrificed at the end of the experiment.

### Western blot analysis

Protein was extracted using RIPA buffer with protease and phosphatase inhibitors. Equal amounts of proteins were electrophoresed and transferred to a nitrocellulose membrane. After immunoblotted with antibodies, the antigen-antibody complexes on the filters were detected by chemiluminescence.

### Immunohistochemistry

Breast tissues and the tumors of MNU-treated rats, xenografted breast tumors of MDA-MB-231 cells in athymic mice were all obtained in a previous study [22]. As previously described [22], immunohistochemical staining was performed with antibodies against TET1 and 5hmC (dilution 1:200) as the primary antibodies. After applied secondary biotinylated antibody, the signal was developed using a modified avidin-biotin complex immunoperoxidase staining procedure according to the manufacturer’s instruction. Stained cells were quantified per high-power field (hpf), and 10 hpf were averaged for each tissue section. At least three sections were analyzed for each sample.

### Transfection of MDA-MB-231 cells

For DNMT1 overexpression, the pcDNA3/Myc-DNMT1 (Plasmid 36,939) plasmid was used. MDA-MB-231 cells were transfected with TET1 siRNA(sc-154,204) for silencing experiments. MDA-MB-231 cells were transfected with Lipofectamine2000 reagent according to the protocol. The cells were collected for the subsequent experiments after 48 h transfection.

### qRT-PCR analysis

Total cellular RNA was isolated using Biozol adopting the manufacturer’s manual. BioRT cDNA First Strand Synthesis Kit, BioEasy SYBR Green I Real Time PCR Kit with specific primers, which were synthesized by Invitrogen were used to quantify the TET1, TET2 and TET3 miRNA transcripts in our study. Each sample was run in triplicate.

### qRT-PCR analysis for miR-34a

Total RNA was extracted. The miRNA first-strand cDNA synthesis kit and miRNA Real-Time PCR Assay kit (Aidlab, Beijing) were applied to quantify the miRNA transcripts. U6 small nucleolar RNA was used as reference. Each reaction sample was run in triplicate. The relative expression level of miRNA was calculated using the comparative CT method (2^−ΔΔCt^).

### Bisulfite modification and methylation-specific PCR (MSP)

The sodium bisulfite modified DNA was used for MSP. The PCR primers used to detect the CpG-methylation of the miR-34a promoter were previously established [16, 17, 22]. Methylated-MSP: forward, 5′-GGTTTTGGGTAGGCGCGTTTC-3′, reverse, 5′-TCCTCATCCCCTTCACCGCCG-3′; unmethylated-MSP: forward, 5′-IIGGTTTTGGGTAGGTGTGTTTT-3′, reverse, 5′-AATCCTCATCCCCTTCACCACCA-3′. The PCR primers used to detect the CpG-methylation of the TET1 promoter were designed with MethPrimer. Methylated-MSP: forward, 5′-TGATAAAATTTTGATATTTTTTTACGT-3′, reverse: 5′-ATAAAACTAAAACTCTACCTTCGCT-3′; unmethylated-MSP: forward, 5′-TGATAAAATTTTGATATTTTTTTATGT3–3′, reverse, 5’AATAAAACTAAAACTCTACCTTCACT-3′. The reactions were carried out as previously [16, 17, 22]. The gel was directly visualized under UV illumination after electrophoresis. Bisulfite template DNA of miR-34a and TET1 were also detected by quantitative PCR (qPCR).

### Chromatin immunoprecipitation(ChIP) assay for TET1 on TET1 promoter

ChIP was performed following the instructions of the EZ-ChIP™ Chromatin immunoprecipitation kit (Millipore). Briefly, MDA-MB-231 cells were treated with 3,6-DHF (20 μM) for 24 h, then washed and crosslinked with 1% formaldehyde for 10 min. The unreacted formaldehyde was quenched with glycine. After sonicated, all samples were chosen with the mean size of DNA fragments maintained at 500 bp. Immunoprecipitation with the indicated antibodies, pre-immune mouse IgG (as a negative control) or anti-RNA Polymerase (as a positive control) was carried out for 24 h with Protein G Agarose. The input (20 μl) and immunoprecipitates were washed and eluted, and the crosslinking was later reversed. After ChIP, qRT-PCR was used to detect the DNA precipitated by the target antibody. Relative data quantification was performed using the 2^−ΔΔCt^ method, and the result was calculated in the form of % Input: %Input = 2^(Ctinput−CtChIP)^ × input dilution factor × 100. The purified DNAs were amplified with the following primer pairs [23]:

TET1 Site-1 Forward(5′-3′):TTGGGAACCGACTCCTCACCT.

TET1 Site-1 Reverse(5′-3′): TCGGGCAAACTTTCCAACTCGC.

TET1 Site-2 Forward(5′-3′): ACGCTGGGCATTTCTGATCCACTA.

TET1 Site-2 Reverse(5′-3′): TATTGTGCAGCTCGTTTAGTGCCC.

TET1 Site-3 Forward(5′-3′): ACTTTGACCTCCCAAAGTGCTGGA.

TET1 Site-3 Reverse(5′-3′):ACCTGAGTGATGCTGAGACTTCCT.

### Dots blot analysis of DNA 5hmC

Genomic DNA samples were extracted from cultured cells. DNA samples were diluted to equal concentrations. After added 0.1 M NaOH, DNA samples were denatured at 95 °C for 5 min, and neutralized with 6.6 M ammonium acetate. The samples were spotted onto a nitrocellulose membrane, then fixed by baking for 30 min at 80 °C. After blocking with 5% skim milk, the membrane was incubated with antibody specific to 5hmC (1:500) followed by incubation with secondary antibody (1:500). The dot signal was visualized with the ECL Plus chemiluminescence assay kit (Fusion FX).

### Statistical analysis

The experimental data are presented as the means ± the standard deviation (SD). The results are from at least three independent experiments. The data were analyzed by one-way ANOVA. Tukey’s test was used for multiple comparisons. Differences were considered statistically significant for *P* < 0.05.

## Results

### 3,6-DHF increases TET1 in breast cancer cells

**Fig. 1:**
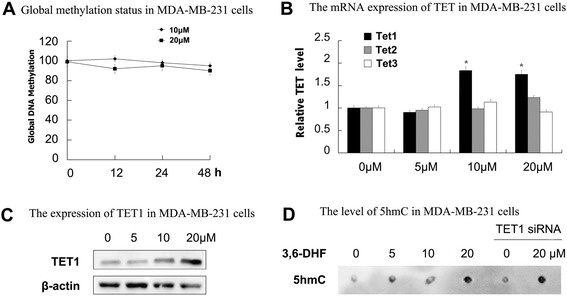
3,6-DHF decreases global DNA methylation levels and promotes the expression of TET1 in breast cancer cells. **a** MDA-MB-231 breast cancer cells were treated with 3,6-DHF (10, 20 μM). The results are expressed as a percentage of vehicle (DMSO)-treated control. **b** Effects of 3,6-DHF treatment (0, 5, 10, and 20 μM) for 24 h on TET1, TET2 and TET3 in MDA-MB-231 cells as detected by qRT-PCR. The data are presented as the mean ± SD (*n* = 3). ^*^ *P* < 0.05 compared with the MDA-MB-231 cells treated with 0 μM 3,6-DHF for 24 h. **c** Western blots showing levels of TET1 in MDA-MB-231 breast cancer cells. **d** Anti-5hmC dot blot for DNA extracted from MDA-MB-231 cells treated with 3,6-DHF

We examined the effect of 3,6-DHF on global DNA methylation in breast cancer MDA-MB-231 cells. As shown in Fig. 1a, after treatment with 10 or 20 μM 3,6-DHF for 24 h, the global DNA methylation showed no significant change. Since the TET family plays potential roles in epigenetic regulation, we detected Tet1, Tet2 and Tet3 mRNA levels in MDA-MB-231 cells. The results (Fig. 1b) indicated that Tet1 mRNA expression was significantly increased after 3,6-DHF treatment for 24 h, while Tet2 and Tet3 showed no notable changes. Western blot detection (Fig. 1c) confirmed that 3,6-DHF increased the level of TET1 and TET1 siRNA blocked the effect(Fig. 3a) in MDA-MB-231 cells. Dot blot analysis demonstrated that 3,6-DHF treatment increased the level of 5hmC(Fig. 1d). There was no detectable effect of knocking down TET1 on global increase of 5hmC level after 3,6-DHF treatment(Fig. 1d).

### 3,6-DHF promotes the expression of TET1 in breast carcinogenesis

**Fig. 2:**
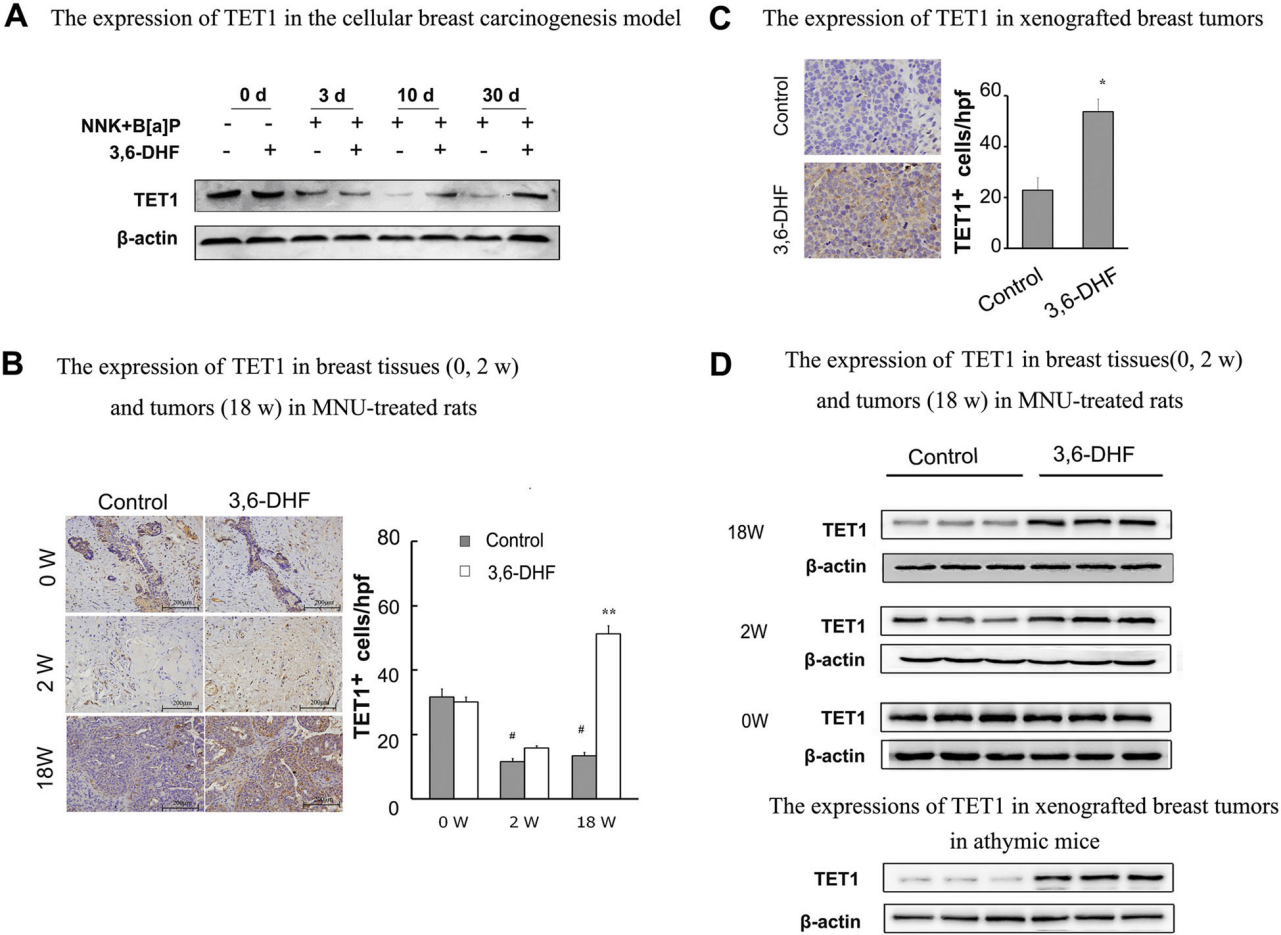
3,6-DHF promotes the expression of TET1 during carcinogens-induced breast carcinogenesis. **a** Western blots showing levels of TET1 in the cellular breast carcinogenesis model. **b** The level of TET1 in breast tissues (0, 2 w) and tumors (18 w) in MNU-treated rats with 3,6-DHF administration (20 mg/kg, i.g.), as detected by immunohistochemistry and western blot **c** Immunohistochemistry of TET1 in xenografted breast tumors in breast tumors in athymic mice. **d** Western blos showing levels of TET1 in breast tissues (0, 2 w) and tumors (18 w) in MNU-treated rats, and in xenografted breast tumors in athymic mice. Immunostaining density was quantified using image J analysis. The data are presented as the mean ± SD (*n* = 3).^*^ *P* < 0.05, ^**^ *P* < 0.01 compared with control. ^#^ *P* < 0.05 compared with 0 W

TET1 and 5hmC down-regulation has been observed more frequently in tumorigenesis [24]. We assessed the TET1 expression in breast carcinogenesis in vitro by chronic exposure to NNK and B[a]P. Our data showed that the levels of TET1 significantly decreased in breast cell carcinogenesis, and 3,6-DHF co-treatment counteracted the decrease of TET1 (Fig. 2a). Then, we detected the expression of TET1 in MNU-treated rats with immunohistochemistry and western blotting. The results (Fig. 2b, d) showed that TET1 levels significantly decreased in breast carcinogenesis in vivo, while 3,6-DHF administration (20 mg/kg, i.g.) could effectively up-regulate the expression of TET1. Furthermore, we found that 3,6-DHF administration promotes the levels of TET1 in xenografted breast tumors derived from MDA-MB-231 cells (Fig. 2c, d).

### 3,6-DHF reactivates the tumor suppressor miR-34a via promoting TET1

**Fig. 3:**
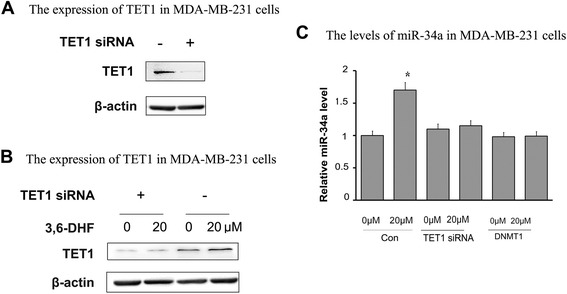
3,6-DHF reactivates the expression of tumor suppressor miR-34a through increasing TET1 level in breast cancer cells. **a** Western blots showing levels of TET1 in MDA-MB-231 cells after transfecting TET1 siRNA. **b** The effect of 3,6-DHF (20 μM) on the levels of TET1 in MDA-MB-231 cells after transfecting TET1 siRNA, detected by Western blotting. **c** The effect of 3,6-DHF (0, 20 μM) on the levels of miR-34a in MDA-MB-231 cells after transfecting TET1 siRNA or pcDNA3/Myc-DNMT1(DNMT1) as detected by qRT-PCR. The data are presented as the mean ± SD (*n* = 3). ^*^ *P* < 0.05, ^**^ *P* < 0.01 compared with the control

**Fig. 4:**
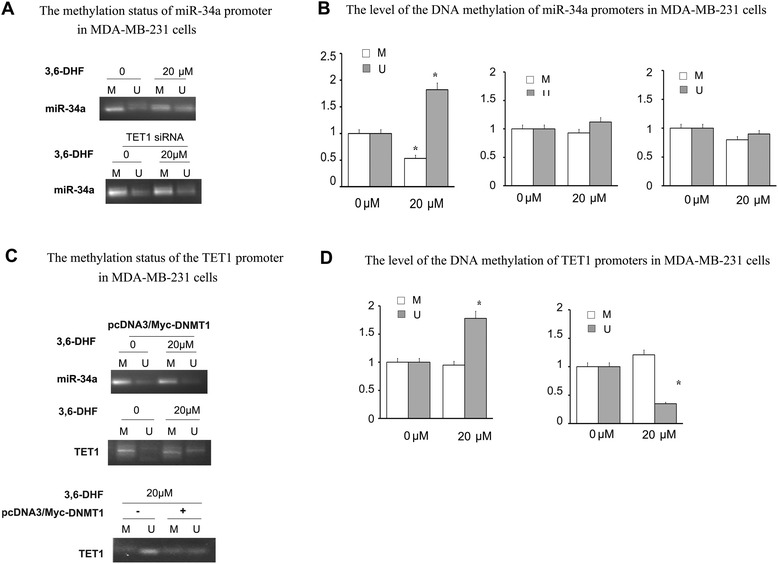
The methylation status of miR-34a and TET1 promoters. **a** The methylation status of miR-34a promoter in MDA-MB-231 cells with 3,6-DHF (20 μM) treatment for 24 h, or transfecting TET1 siRNA before 3,6-DHF (20 μM) treatment for 24 h. or transfecting pcDNA3/Myc-DNMT1 before 3,6-DHF (20 μM) treatment for 24 h. **b** The level of the DNA methylation of miR-34a promoters in MDA-MB-231 cells as determined by qPCR according to Fig. 4a. **c** The methylation status of the TET1 promoter in MDA-MB-231 cells after 3,6-DHF (20 μM) treatment for 24 h, or transfecting of pcDNA3/Myc-DNMT1 before 3,6-DHF (20 μM) treatment. **d** The level of the DNA methylation of TET1 promoters in MDA-MB-231 cells as determined by qPCR according to Fig. 4c. Methylation status was detected by MSP; methylation levels are also detected with qPCR. M: methylated; U: unmethylated. The data are presented as the mean ± SD (*n* = 3). *P < 0.05 compared with the control or compared with 0 μM

Our previous study revealed that 3,6-DHF increases the level of miR-34a in breast cell carcinogenesis and breast cancer cells. However, the mechanism is unclear. We blocked TET1 expression by siRNA to evaluate the role of TET1 in 3,6-DHF-induced up-regulation of miR-34a in MDA-MB-231 cells (Fig. 3a, b).The results showed that inhibition of TET1 significantly suppresses the effects of 3,6-DHF on miR-34a (Fig. 3c). MSP assays showed that 3,6-DHF decreases the methylation of the miR-34a promoter, and that TET1 inhibition could counteract the effect of 3,6-DHF on the miR-34a promoter (Fig. 4a, b). These data suggests that 3,6-DHF up-regulates miR-34a by increasing TET1 expression and thus demethylation of miR-34a promoter.

### 3,6-DHF improves the level of TET1 by repressing DNMT1

**Fig. 5:**
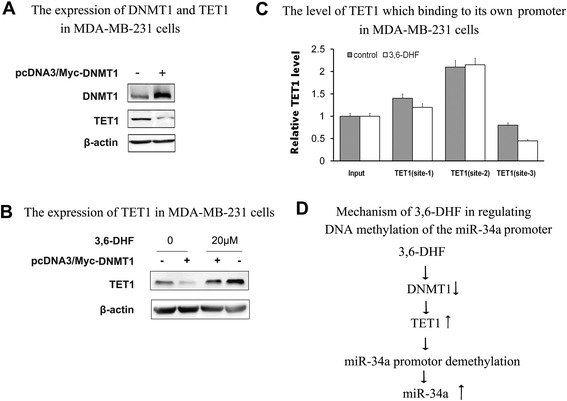
3,6-DHF improves the expression of TET1 by repressing DNMT1 activity. **a** Western blots showing the levels of DNMT1 and TET1 in MDA-MB-231 cells after transfecting pcDNA3/Myc-DNMT1. **b** The effect of 3,6-DHF (20 μM) on the levels of TET1 after transfecting pcDNA3/Myc-DNMT1, detected by western blot analysis. **c** The level of TET1 binding to its own promoter in MDA-MB-231 cells as determined by a ChIP assay with anti-TET1 antibody followed by qPCR; Site-3 is a negative control. The data are presented as the mean ± SD (*n* = 3). **d** Flow chart illustrating mechanism of 3,6-DHF in regulating DNA methylation of the miR-34a promoter

Our previous study observed that 3,6-DHF is an effective DNMT1 inhibitor and decreases DNMT activity in MDA-MB-231 cells [22]. In this study, we evaluated the effect of DNMT1 on 3,6-DHF-induced promotion of TET1 by transfecting DNMT1 plasmids in MDA-MB-231 cells. As expected, over-expression of DNMT1 significantly down-regulated TET1 and reduced the promotional effect of 3,6-DHF on TET1 (Fig. 5a, b). MSP detection indicated that DNMT1 over-expression inhibits the effect of 3,6-DHF on methylation of the TET1 promoter (Fig. 4c, d). The results also showed that DNMT1 over-expression significantly reduces 3,6-DHF activation of miR-34a (Fig. 3c) and inhibits the demethylation effect of 3,6-DHF on the miR-34a promoter (Fig. 4a, b). Because TET1 may bind to its own promoter region directly, we analyzed whether 3,6-DHF influenced the autoregulation of TET1. ChIP assays showed that 3,6-DHF did not increase the binding of TET1 on its own promoter (Fig. 5c). These findings indicate that 3,6-DHF increases TET1 expression by demehylation of the TET1 promoter partially through the inhibition of DNMT1.

## Discussion

Investigate the factors that relate to carcinogenesis may contribute to strategies for cancer treatment and prevention [25]. As epigenetic aberrations occur and initiate events in tumorigenic processes, epigenetic treatment is a promising strategy for cancer prevention [26]. Some phytochemicals are shown to modulate epigenetic modifications. Several phytochemicals such as resveratrol [27], curcumin [28], tea phenols [29], genistein [30] and sulforaphane [31] inhibit DNA methyltransferases and alter DNA methylation of some genes. Phytochemicals, such as EGCG [32], organosulfur compounds [33] and resveratrol [34], have critical roles in histone acetylation or deacetylation. Elagitannins, EGCG, genistein, indole-3-carbinol and resveratrol also have effects on miRNAs oncogenic relating with carcinogenesis [35]. In our research, we observed that 3,6-DHF could reverse the global down-regulation of miR-34a in breast carcinogenesis by regulating the miR-34a promoter methylation. Regulation of the cytosine methylation status of promoters could contribute to the epigenetic control of 3,6-DHF in carcinogenesis. This finding prompted us to further study the mechanism of 3,6-DHF in regulating DNA methylation of the miR-34a promoter.

Considerable attention has been focused recently on the crucial role of DNA methylation in tumorigenesis, and demonstrates its potential as a disease biomarker and therapeutic cancer target. DNMT1 is the most abundant DNMT which maintains the DNA methylation pattern. The expression levels of DNMT1 are reportedly elevated in various cancers [36]; reduction of DNMT1 also blocks tumorigenesis [37]. In our previous research, we found 3,6-DHF inhibits the activity of DNMT1, and now we further confirmed the effect of 3,6-DHF on DNMT1 by expression of DNMT1 plasmids. DNMT1 over-expression blocked the effect of 3,6-DHF on increasing miR-34a mRNA and miR-34a promoter demethylation, suggesting that 3,6-DHF could reactivate tumor suppressor genes silenced by promoter methylation during tumorigenesis by repressing DNMT1 activity.

TET protein expression and its dominant enzymatic product (5hmC) are markedly reduced in breast tumors [38]. Researchers found that decreased 5hmC or TET levels have a close correlation with robust tumor growth and metastasis. Increasing TET activity could be a useful strategy in cancer treatment [39]. For example, vitamin C has the role of increasing DNA demethylation through enhancing TET activity in cancer cells [40]. In our research, we found that 3,6-DHF treatment increased TET1 level in MDA-MB-231 cells, and had no effect on TET2 and TET3. By immunohistochemistry, we found that the level of TET1 significantly decreased during carcinogen-induced breast carcinogenesis in MCF10A cells and rats, and that 3,6-DHF administration could effectively up-regulate the expression of TET1*.* 3,6-DHF administration also promoted the levels of TET1 and 5hmC in xenografted breast tumors derived from MDA-MB-231 cells, confirming the effect of 3,6-DHF on TET1. TET1 inhibition with siRNA in MDA-MB-231 cells blocked the effect of 3,6-DHF on increasing miR-34a mRNA and miR-34a promoter demethylation, suggesting that the increase of TET1 could be one of the mechanisms of breast cancer prevention by 3,6-DHF. Furthermore, DNMT1 over-expression in part blocked the effect of TET1 on miR-34a by TET1 promoter demethylation. Thus we can conclude that 3,6-DHF inhibits DNMT1 activity, modulates the imbalance of DNA methylation and demethylation status, increases TET1 expression, re-expresses miR-34a, and as a consequence, prevents breast carcinogenesis. MiR-34a levels are not only determined by transcriptional regulation, but also by processes relating to miRNA biogenesis. We will continue this interesting research in further studies.

## Conclusions

Our study showed that 3,6-DHF increases TET1 expression during carcinogenesis and up-regulates miR-34a level by regulating the methylation status of DNA.

## Abbreviations

3,6-DHF: 3,6-dihydroxyflavone
5hmC: 5-hydroxymethylcytosine
B[a]P: benzo[a]pyrene
BC: Breast cancer
ChIP assay: Chromatin immunoprecipitation assay
DNMTs: DNA methyltransferases
MSP: Bisulfite Modification and Methylation-Specific PCR
NNK: 4-(methylnitrosamino)-1-(3-pyridyl)-1-butanone
SAM: Methyl donor S-adenosyl-methionine
TET: Ten-eleven translocation
